# PATTERN RECOGNITION AND INNOVATION OF HOME-BASED CARE IN CHINA: FROM THE PERSPECTIVE OF MULTINATIONAL COMPARISON

**DOI:** 10.1093/geroni/igad104.1599

**Published:** 2023-12-21

## Abstract

The development trend of family structure’s miniaturization and nucleation in China and the overlapping occurrence of population aging and associated frailty and morbidities have created an urgent need for innovation in the home care service model. In this paper, home-based care is regarded as a dynamic pension method. Based on the analysis of dynamic changes in population structure and family structure, this paper uses multi-stage microsimulation technology to scientifically predict the evolution of the older adult population’s distribution state and multi-health state. This approach leads to early identification of the risks of various home-based care models in the process of family structure changes. Based on this, with reference to the home-based care model cases in China, Japan, Europe, and the United States, this paper proposes the innovation of the multi-health state and the “ integrated care at home” model for households with an aging resident and brings up the optimization plan of home-based care service for accelerating the aging process. Implications for policies and practices to support family members to realize this optimized home care model will be discussed.

